# Atlas and Epitome of Gynecology

**Published:** 1901-03

**Authors:** 


Atlas and Epitome of Gynecology, by Dr. Oskar Schaeffer, Private docent of Obstetrics and Gynecology in the University of Heidelberg. Authorized translation from the Second Revised and Enlarged German Edition, Edited by Richard C. Norris, A. M., M. D., Surgeon-in-Charge, Preston Retreat, Philadelphia; Gynecologist to the Methodist Episcopal Hospital and to the Philadelphia Hospital; Consulting Gynecologist to the Southwestern Dispensary and Hospital for Women and Children; Lecturer on Clinical and Operative Obstetrics, Medical Department, University of Pennsylvania. With 207 Colored Illustrations on 90 Plates, and 62 Illustrations in the Text. Pp. 272; price, $3.50. Philadelphia: W. B. Saunders & Co., 1900.

This work is intended to occupy the position midway between the quiz compend and the more pretentious works on gynecology. The student and general practitioner will appreciate the concise explanatory text and the well executed illustrations. The attempt to imitate in appearance the fresh specimen is a failure. The coloring is exaggerated, as is generally the case, but the common sense displayed in the choice of parts illustrated and the practical arrangement of the subject matter make the work a very valuable one.

